# Loss of Notch signalling induced by Dll4 causes arterial calibre reduction by increasing endothelial cell response to angiogenic stimuli

**DOI:** 10.1186/1471-213X-8-117

**Published:** 2008-12-16

**Authors:** Rui Benedito, Alexandre Trindade, Masanori Hirashima, Domingos Henrique, Luis Lopes da Costa, Janet Rossant, Parkash S Gill, António Duarte

**Affiliations:** 1 Centro Interdisciplinar de Investigação em Sanidade Animal, Faculdade de Medicina Veterinária, Technical University of Lisbon, Lisboa, Portugal; 2 Instituto Gulbenkian de Ciência, Oeiras, Portugal; 3 Sakaguchi Laboratory of Developmental Biology, School of Medicine, Keio University, Tokyo, Japan; 4 Instituto de Medicina Molecular, Faculdade de Medicina da Universidade de Lisboa, Lisboa, Portugal; 5 Department of Pathology, University of Southern California Keck School of Medicine, Los Angeles, CA, USA; 6 Program for Developmental Biology, Hospital for Sick Children, Toronto, Canada; 7 Max Planck Institute for Molecular Biomedicine, Muenster, Germany

## Abstract

**Background:**

In the vascular system, Notch receptors and ligands are expressed mainly on arteries, with Delta-like 4 (Dll4) being the only ligand known to be expressed early during the development of arterial endothelial cells and capillaries. *Dll4 *null embryos die very early in development with severely reduced arterial calibre and lumen and loss of arterial cell identity.

**Results:**

The current detailed analysis of these mutants shows that the arterial defect precedes the initiation of blood flow and that the arterial *Dll4*^-/- ^endothelial cells proliferate and migrate more actively. *Dll4*^-/- ^mutants reveal a defective basement membrane around the forming aorta and increased endothelial cell migration from the dorsal aorta to peripheral regions, which constitute the main causes of arterial lumen reduction in these embryos. The increased proliferation and migration of *Dll4*^-/- ^endothelial cells was found to coincide with increased expression of the receptors VEGFR-2 and Robo4 and with downregulation of the TGF-β accessory receptor Endoglin.

**Conclusion:**

Together, these results strongly suggest that Notch signalling can increase arterial stability and calibre by decreasing the response of arterial endothelial cells to local gradients of pro-angiogenic factors like VEGF.

## Background

The initial steps of embryonic blood vessel formation (vasculogenesis) involve differentiation and aggregation of mesodermal-derived endothelial precursors (angioblasts) to generate the primary vascular plexus. Blood vessels then grow and remodel into a mature network of hierarchically organized vessels, in a process designated as angiogenesis. During this remodelling, endothelial vessels differentiate into arteries and veins, grow, sprout, split and fuse according to a combination of autocrine signalling and cues provided by other tissues of the embryo [1].

The formation of the initial vascular plexus is highly dependent on vascular endothelial growth factor (VEGF-A) and its receptors, VEGFR-1 and VEGFR-2, since mouse embryos lacking these genes display very early vascular defects and die between 8.5–9.5 dpc [2-4]. Notably, *VEGF *haploinsufficiency causes embryonic lethality. VEGFR-2 is thought to be the major mediator of the VEGF signalling since *VEGFR*-2^-/- ^embryos fail to form blood islands or organized blood vessels [3], whereas *VEGFR-1*^-/- ^embryos form blood vessels with an excess of endothelial and haemangioblast progenitors [4,5]. Furthermore, deletion of the VEGFR-1 tyrosine kinase domain does not affect vascular development [6]. In some contexts VEGFR-1 seems to work as a biological sink to VEGF, antagonizing VEGFR-2 mediated signalling [7,8]. Through activation of VEGFR-2, VEGF induces phosphorylation of several proteins which in turn regulate endothelial cell permeability, proliferation, migration and survival. During later vessel remodelling, other ligands become increasingly important and the disruption of the signalling induced by angiopoietins, ephrins, TGF-β, slits or netrins causes specific vascular defects that often leads to early embryonic death [9,10].

The Notch pathway is also involved in the regulation of vascular development as the single gene deletion of *Notch1 *or combined deletion of *Notch1 *and *Notch4 *results in severe defects in early arterial development [11]. The Notch receptors can bind five known ligands from the Delta (Dll1, Dll3 and Dll4) and Jagged (Jagged1 and Jagged2) families. The Dll4 ligand is the only canonical Notch ligand strongly expressed in arterial endothelial cells at early stages of development [11,12]. Beside *VEGF, Dll4 *is the only other known vascular-related haploinsufficient lethal gene, indicating how critical its dosage levels are for vascular development [13-15]. We have previously shown that the majority of the *Dll4*^+/- ^embryos die at 10.5 dpc in the CD1 genetic background, whereas all the *Dll4*^-/- ^embryos die at 9.5 dpc with severely reduced dorsal aortae. Furthermore, *Dll4*^-/- ^aortic endothelial cells lose the expression of the arterial-specific genes *Efnb2 *and *Gja4 *(*Cx37*), and ectopically express the vein marker *Ephb4*, one of the receptors for the arterial ephrinB2 ligand [15].

In order to understand the cause of this arterial calibre and lumen reduction, a stepwise analysis of the early vascular development of *Dll4*^-/- ^embryos was carried out.

## Methods

### Mice

All procedures complied with the standards for care and use of animal subjects as stated in the Guide for the Care and Use of Laboratory Animals (Institute of Laboratory Animal Resources, National Academy of Sciences of the U.S.A.) and were approved by the institutional committee for the ethical use of laboratory animals of the Faculdade Medicina Veterinaria (project POCTI 48677). Embryos were obtained by the intercross of *Dll4*^+/- ^mice in the CD1 genetic background as described previously [15]. These heterozygous mice were kept in the CD1 genetic background due to the higher lethal haploinsufficiency in the other tested genetic backgrounds (almost 100%). The embryonic age was initially determined by the date of the formation of the copulation plug and confirmed by the number of somites.

### X-Gal staining, immunofluorescence and in situ hybridization

Whole-mount immunohistochemistry and X-gal staining were carried out by standard techniques [16]. For sections, after fixation in 4% paraformaldehyde (PFA), embryos were cryoprotected in 15% sucrose, embedded in 7.5% gelatine and cryosectioned at 12 μm. Antibodies against PECAM-1 (Pharmingen), α SMA (Sigma), SM22α (Abcam), β-catenin (Sigma), Fibronectin (Sigma), Endoglin (Pharmingen), Laminin (Abcam) and CollagenIV (Chemicon) were used as primary antibodies and species-specific conjugated to Alexa Fluor (Molecular Probes) were used as secondary antibodies.

*In situ *hybridization of cryostat sections with digoxigenin labelled riboprobes *Dll4 *(full-length); *Flk1 *[17]; *Flt1 *[4]; *Notch1 *(a 2168 bp BamHI-EcoRV fragment of the NM_008714 cDNA clone, starting at nucleotide 4205 and ending at nucleotide 6373); *Hey1 *[18], *VEGFA *[19], was essentially done as previously described [20]. After overnight incubation with anti-Dig-AP (1:2000, Roche) the signal was developed with Fast Red solution (Roche) or BM purple (Roche). The double *in situ *hybridization was done with the *Dll4 *riboprobe labelled with fluorescein and the *Notch1 *with digoxigenin. For the amplification of the *Dll4 *signal was used the anti-Fluo-POD (1:100, Roche) and the FITC-tyramide from the TSA-Plus Fluorescein System (Perkin Elmer). The *Notch1 *signal was developed after the *Dll4 *signal as for the single in *situ *hybridizations. Fluorescence images were acquired on a Leica DM5000B microscope (sections) or an Olympus SZX12 stereomicroscope (wholemount). Confocal images were acquired using a Leica Spectral AOBS microscope. All the images were processed on Adobe Photoshop 9.0.

### BrdU and TUNEL labelling

BrdU labelling was achieved by a 2 hour BrdU pulse (100 ug BrdU/g body weight, intraperitoneally) before fixation in 4% PFA. Cryosections from the fixed embryos were treated in 50% formamide/1 × SSC, pH = 7 for 1 hour at 65°C and HCl (7.5%) for 30 minutes at 37°C and incubated overnight with mouse anti-BRDU (1:1000, Sigma). To visualize the nucleus a15 minute incubation in DAPI solution followed by a brief fixation in 4% PFA was performed before the DNA denaturation. Endothelial anti-PECAM-1 immunostaining was performed also before the BrdU immunostaining. In this experiment we used consecutive cryosections from all the anterior-middle region of the embryos (2 embryos from each genotype). From each cryosection a 3 channel picture was taken and used to score the number of total and proliferating aortic endothelial cells.

To detect apoptotic endothelial cells on cryosections, anti-PECAM-1 and DAPI staining after TUNEL labelling and staining with the fluorescein *in situ *cell death detection it (Roche) was performed. Cell counting was carried out as described for the BrdU staining.

### Quantitative PCR

Total RNA was extracted from whole embryos, after removal of the yolk sac, with the RNeasy Mini Kit (Qiagen). cDNA synthesis was done using the Superscript III First Strand Synthesis kit (Invitrogen). In two different experiments embryos were obtained from two different litters (n = 4 for each genotype). In each experiment, for each analysed gene, 6 separate real time PCR (RT-PCR) reactions were carried out, in triplicates, using SYBR Green Mixes and ABI PRISM 7300 (Applied Biosystems). Gene expression was normalised to *β-actin*. Used Primer sequences are as follows: *β-actin *5'-TGTTACCAACTGGGACGACA-3'and 5'-GGGGTGTTGAAGGTCTCAAA-3';*VEGFR2 *5'-GGCGGTGGTGACAGTATCTT-3' and 5'-GAGGCGATGAATGGTGATCT-3' *VEGFR1 *5'-GACCCTCTTTTGGCTCCTTC-3'and 5'-CAGTCTCTCCCGTGCAAACT-3' ; *VEGFA *5'-GGAGAGCAGAAGTCCCATGA-3' and 5'-ACACAGGACGGCTTGAAGAT-3'; *Hey1 *5-CACCTGAAAATGCTGCACAC-3' and 5'-ATGCTCAGATAACGGGCAAC-3'; *Hey*2 5'-TGCCAAGTTAGAAAAGGCTGA-3' and 5'-CACTCTCGGAATCCAATGCT-3'; *Cx37 *5'-ACGGTCGTCCCCTCTACCT-3' and 5'-GTCGAGTGTTCCTGGACCTG-3'; *Endoglin *5'-CACACCTCCCAAGGACACAT-3' and 5'-TGGAGTCCCAGAAAGTCAGG-3'; *Robo4 *5'-CGAGGACGCCATTCTAAAAC-3' and 5'-CCAATCCCAGCCGACTACTA-3' and *PECAM *5'-GTGGTCATCGCCACCTTAAT-3' and 5'-GGCTTCCACACTAGGCTCAG-3'.

## Results

### Reduced arterial calibre in *Dll4*^-/- ^embryos is determined before the onset of blood flow and is not caused by defects in angioblast differentiation

**Figure 1:**
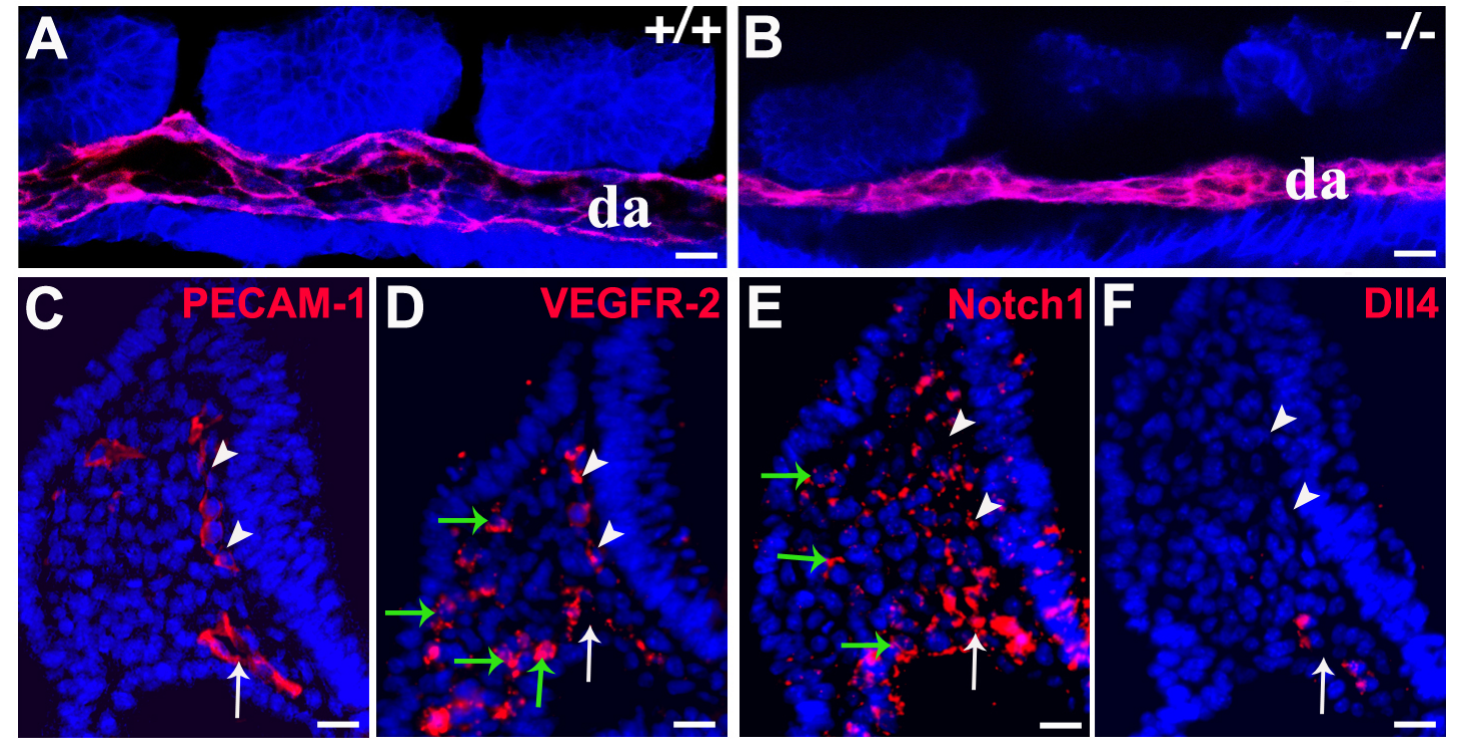
**The dorsal aortae calibre and lumen reduction in the *Dll4*^-/- ^embryos is not caused by blood flow or angioblast defects**. (***A, B***) Confocal imaging analysis of 8 ss *Dll4*^+/+ ^and *Dll4*^-/- ^embryos showing in red the dorsal aortae (da) endothelial cells (labelled with anti-PECAM-1) and in blue all the other cells from the adjacent tissues (labelled with anti-β-catenin). (***C-F***) Anti-PECAM-1 or fluorescent *in situ *hybridization on consecutive cryosections from a *Dll4*^+/+ ^8 ss embryo, showing that VEGFR-2 and Notch1 are expressed in the endothelial cells (PECAM-1^+^) and in the mesoderm cells from the lateral plate (green arrows), where angioblasts arise. The *Dll4 *mRNA is not detected on the angioblasts or on the primordial anterior cardinal vein (white arrowheads) but only on aortic endothelial cells (white arrows). (Scale Bars: 15 μm).

The *Dll4 *gene starts to be expressed in the embryo at the 2-somite stage in the endothelium of the developing heart and dorsal aortae [15]. As the development of the dorsal aortae is coincident with heart development and the initiation of blood flow, we asked if the early arterial calibre and lumen reduction seen in the *Dll4*^-/- ^embryos could be related to impaired haemodynamic forces, which are known to influence arterial identity and remodelling [21]. Confocal imaging of the dorsal aortae of 8-somite stage (ss) embryos shows that in *Dll4*^-/- ^embryos the arterial calibre and lumen is already significantly reduced before the initiation of heart beat and blood flow, which begins around 10–11 ss (Fig. 1A, B). Together with previous observations [22], these results suggest that the initial regulation of blood vessel size is an intrinsic property of early endothelial cells.

The reduction of the arterial calibre could arise from either defects in the maturation of the dorsal aorta once formed or from abnormal differentiation and impaired migration of angioblasts from the lateral plate mesoderm to the midline of the embryo. Angioblasts are endothelial progenitors and express high levels of VEGFR-2 and no detectable levels of PECAM-1 [22]. *In situ *hybridization with probes for the *Notch1 *and *Dll4 *mRNAs shows that Dll4 is not expressed in cells from the lateral plate mesoderm, where angioblasts (VEGFR-2^+^/PECAM^-^) are located (Fig. 1C, D, E and 1F), and only becomes expressed after initiation of aorta formation. This indicates that the reduction of the arterial calibre observed in the *Dll4*^-/- ^embryos is not caused by defects in early angioblast differentiation or migration to the midline, since *Dll4 *is only expressed at the aortic endothelium at this stage.

### *Dll4*^-/- ^arterial endothelial cells display abnormal morphology and a defective basement membrane

**Figure 2:**
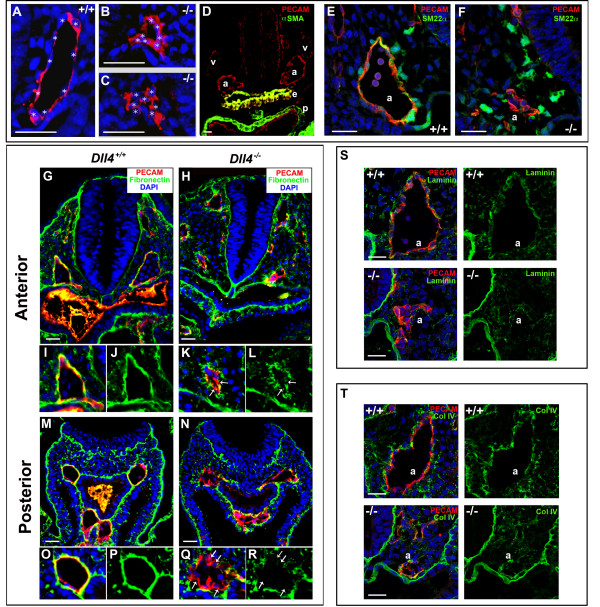
**The *Dll4*^-/- ^arterial endothelial cells have an abnormal morphology, become more aggregated and display a defective basement membrane**. (**A-C**) Anti-PECAM-1 staining of 8 ss embryo cryosections showing the morphology of *Dll4*^+/+ ^and *Dll4*^-/- ^dorsal aortae endothelial cells. The white stars label the DAPI stained endothelial nuclei. *Dll4*^-/- ^endothelial cells are more aggregated and fail to develop a stretched spindle-like form which leads to a reduced (**B**) or absent aortic lumen (**C**). (**D**) Anti-PECAM-1 (red) and anti-αSMA (green) staining of 16 ss cryosection of a *Dll4*^+/+ ^embryo showing the absence of arterial (a) and venous (v) endothelium associated smooth muscle cells. Note the strong αSMA staining in the endoderm (e) and pericardium (p). (**E, F**) Both control and mutant dorsal aortae (a) are surrounded by neural crest derived smooth muscle cell progenitors with high amounts of Sm22α protein (in green). (**G-R**) Anti-PECAM-1 (red), anti-fibronectin (green) and DAPI (blue) staining of cryosections from the anterior (**G-L**) and posterior (**M-R**) region of 11 ss *Dll4*^+/+ ^and *Dll4*^-/- ^embryos. Figures **I-L **and **O-R **are close ups of the upper left dorsal aortae and show the defective basement membrane (green) in both the anterior and posterior regions of the *Dll4*^-/- ^aortae. In the mutants many endothelial cells are not in direct contact with the underlying, fibronectin-rich, extracelular matrix (arrows). (**S, T**) At this stage of development the aortic (a) basement membrane is relatively poor in laminin (S, control) and collagenIV (T, control) when compared with fibronectin (J and P). The reduced *Dll4*^-/- ^dorsal aortae have even less and more irregular laminin and collagenIV coverage. (Scale Bars: 30 μm).

To study the nature of *Dll4*^-/- ^endothelial cell phnotype we analysed endothelial morphology and extracellular matrix integrity in embryos with 8–11 somites, the stage immediately before the onset of circulation and when the phenotype starts to become more pronounced. The analysis of transverse sections labelled with the endothelial marker PECAM-1 revealed that the *Dll4*^-/- ^aortic endothelial cells, in the anterior and middle region of the embryo, are frequently more aggregated and do not acquire a spindle-like shape (Fig. 2A, B and 2C). The majority of these cells display a rounder morphology which is characteristic of incompletely differentiated or loosely attached endothelial cells. Interestingly, fibronectin, laminin and collagenIV immunostaining further revealed that these cells have a severely defective basement membrane in both the anterior and posterior (presomitic) dorsal aortae (Fig. 2G-T). Whereas in a normal developing dorsal aorta there is accumulation of a thick layer of surrounding fibronectin, in the *Dll4*^-/- ^embryos the endothelial matrix is very irregular and some of the arterial endothelial cells are not in direct contact with it. These defects occur both in the anterior and presomitic domains of the dorsal aortae, whereas the arterial calibre reduction occurs only in the anterior domain. This suggests that the defects observed in basement membrane integrity are not sufficient to explain the reduction in the arterial calibre seen in the *Dll4*^-/- ^embryos.

### *Dll4*^-/- ^aortic defects precede smooth muscle development

Besides the formation of the basement membrane, another biological process that confers stability and cohesion to the developing arteries is the recruitment of pericytes and smooth muscle cells. Immunostaining for alpha smooth muscle actin (αSMA) (Fig. 2D) and *in situ *hybridization for *Pdgfrb *(data not shown) on *Dll4*^+/+ ^embryonic sections at this developmental stage, showed that there is virtually no differentiated smooth muscle cells or pericytes associated with the endothelium of these primordial arteries. To further investigate a possible function of *Dll4 *in arterial smooth muscle cell recruitment we did imunostaining for Sm22α a gene highly expressed by neural crest derived smooth muscle cells and progenitors. We detected Sm22α in several cells surrounding the control and mutant aortic endothelium but just some Sm22α positive cells were found immediately adjacent and adherent to the aortic endothelial cells (Fig. 2E, F). Most of the mural cells Sm22α+ in contact with the endothelial cells showed a round shape and were not completely attached to it. In fact we detected less Sm22α+ cells in contact with the *Dll4*^-/- ^aortas, although this can be related to the reduced arterial calibre and available endothelial cell surface. We also analysed Sm22α staining in the pre-somitic region, where *Dll4*^-/- ^and control aortae have a similar calibre, but at this level we could not observe Sm22α+ cells surrounding the aorta of either control or mutant embryos. These results suggest that the arterial phenotypic consequences of *Dll4 *loss of function precede the existence of fully differentiated supporting cells in the arteries.

### Loss of the Notch ligand Dll4 increases endothelial cell proliferation and migration

**Figure 3:**
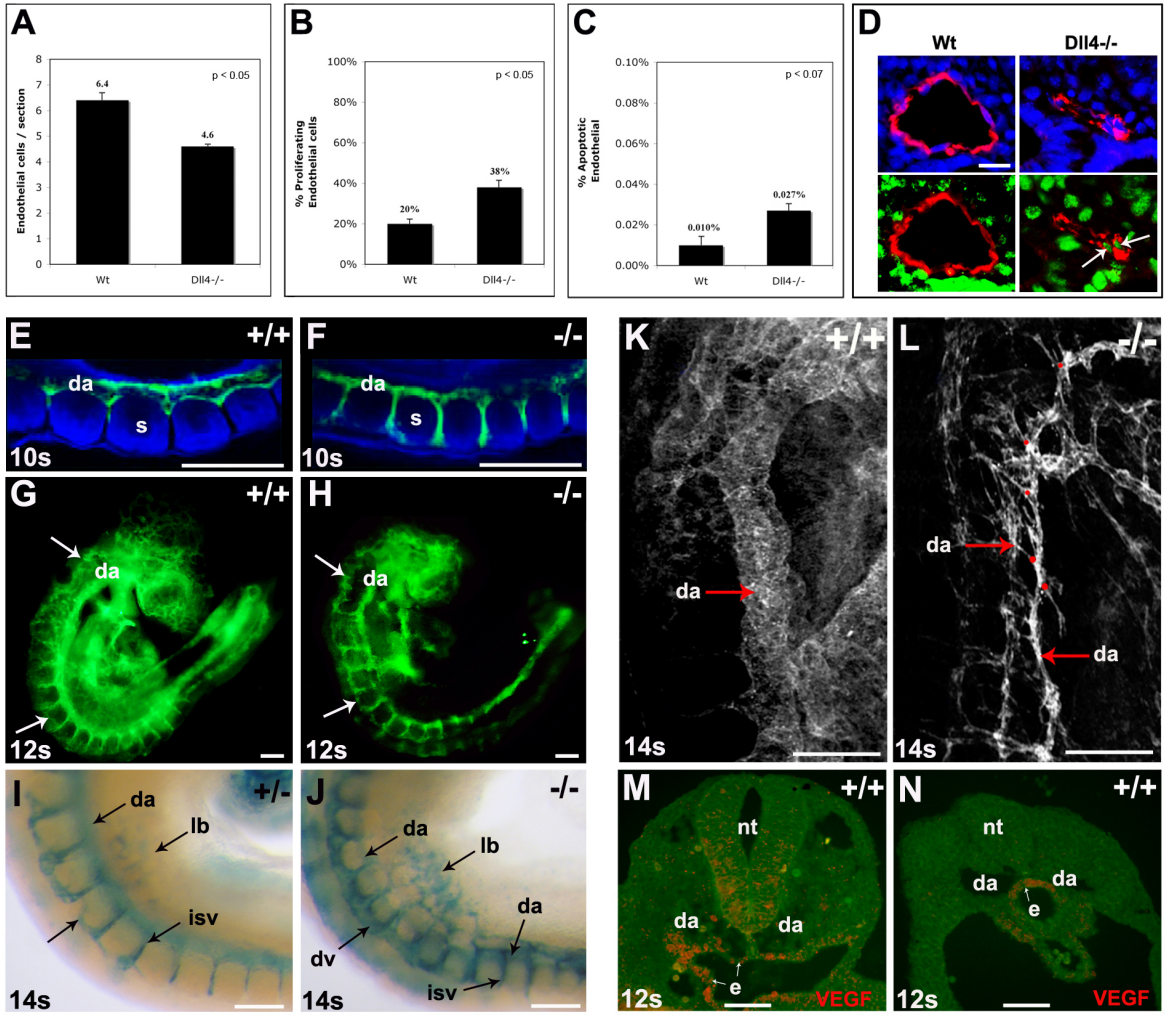
**Increased proliferation, sprouting and migration of *Dll4*^-/- ^arterial endothelial cells**. **(A-C)** Quantification of the endothelial cell number per aortic section (A), percentage of proliferating endothelial cells (B), and percentage of apoptotic endothelial cells (C) in the aortae of 8ss Wt and *Dll4*^-/-^ embryos (n=2 for each genotype, 30 sections per embryo). **(D)** Representative images of the sections used for quantification. Endothelial cells are shown in red (anti-PECAM-1), nuclei in blue (DAPI) and proliferating cells in green (anti-BRDU). The proliferating endothelial cells are also indicated (arrows). **(E-J)** Whole-mount embryos labelled with anti-PECAM-1 (E-H, green) or X-gal (I, J) showing the increased migration of intersomitic vessel (isv) *Dll4*^-/- ^endothelial cells. (E, F) Confocal imaging of the somitic region of 10ss embryos showing that in *Dll4*^-/- ^embryos (F) the aortae endothelial cells invade the intersomitic space earlier and migrate extensively, causing the calibre reduction of the dorsal aortae (da). (G, H) At 12ss the endothelial cells start to accumulate in the dorsal domain (arrows) of the *Dll4*^-/- ^embryos. (I, J) The X-gal staining of 14ss embryos shows the accumulation of endothelial cells (blue) in the dorsal region of the *Dll4*^-/- ^first anterior-middle somites (J), forming a large dorsal vessel (dv). The *Dll4*^-/- ^cells also migrate extensively to the primordial limb buds (lb). **(K, L)** Confocal imaging of 14ss wholemount embryos labelled with anti-PECAM-1. The *Dll4*^-/- ^dorsal aortae (da) are thinner and show extensive sprouting (red dots). **(M, N)** In situ hybridization for *VEGF* mRNA on cryosections of a 12ss embryo shows that in the anterior region (M), VEGF is expressed by both the neural tube (nt) and endoderm (e) cells, but in the posterior (pre-somitic) region (N) is only expressed by the endoderm cells. Error bars indicate *s.e.m.* (Scale Bars: D, 25mm; E-J, 150mm; K-N, 100μm).

To further characterize the *Dll4*^-/- ^endothelial cell behaviour we analysed the *in vivo *proliferation and migration of endothelial cells. To detect the primary defect the analysis of the aortic endothelial cell number and proliferation rate was done with *Dll4*^+/+ ^and *Dll4*^-/- ^embryos with 8 somites. Each embryo was serially sectioned and immunostained for PECAM-1 and BrdU. At this early stage of development there are 28% less endothelial cells in the *Dll4*^-/- ^aortae compared to the control dorsal aortae (Fig. 3A), and as development proceeds this difference becomes more pronounced (data not shown). Unexpectedly, however, the proliferation rate of aortic *Dll4*^-/- ^endothelial cells is almost two-fold higher (Fig. 3B, D). This apparent contradiction could be explained by either increased apoptosis or increased outward migration of the aortic *Dll4*^-/- ^endothelial cells. To test the first hypothesis, we performed TUNEL analysis, which showed that although there is an increase of endothelial cell apoptosis, its rate is too low to counterbalance the increase in endothelial cell proliferation (Fig. 3C).

The sequential analysis of the intersomitic vessel development from 10 to 14 ss embryos showed that *Dll4*^-/- ^aortic endothelial cells migrate earlier and faster through the intersomitic space and accumulate in excessive numbers in the region of dorsal anastomotic vessel formation (Fig. 3E–J). Every time a somite forms in the posterior region a new gradient of angiogenic factors is established within the newly formed intersomitic space boundaries, which attracts the endothelial cells. Therefore, the migration of *Dll4*^-/- ^endothelial cells is more advanced in the first than in the last somites. Consequently, the mutant dorsal aortae calibre is also much more reduced in the anterior somites and increases gradually until the most posterior somites and presomitic mesoderm, where its calibre is normal (Fig. 3J). To further investigate how these embryonic tissues regulate this gradual endothelial cell migration, we performed *in situ *hybridization with a probe to the *VEGF-A *mRNA on embryonic sections. In the anterior and middle regions of the embryo, the neural tube and the endoderm express high levels of *VEGF-A*, but in the presomitic mesoderm, where dorsal aortae do not sprout, *VEGF-A *mRNA was only detected in the endoderm, close to the dorsal aortae (Fig. 3M, N). This indicates that the neural tube, and not only the somites, can be a regulator of intersomitic vessel sprouting and dorsal growth. The results suggest that in the absence of angiogenic gradients secreted by the somites and the neural tube (as in the presomitic mesoderm), the *Dll4*^-/- ^endothelial cells remain associated with the dorsal aortae and form a normal arterial lumen, even though they have a defective basement membrane as described above.

This increased migration of arterial endothelial cells also occurs towards the region of the developing limb bud, where an accumulation of *Dll4*-lacZ ^+ ^endothelial cells can be seen (Fig. 3I, J). In the anterior asomitic region of the embryo there is also an increased accumulation of endothelial cells in a dorsal region where the primordial anterior cardinal vein arises and develops. Confocal analysis shows increased but restricted sprouting of endothelial cells from the dorsal aortae, since we did not observe further extension and somite invasion of these sprouts (Fig. 3K, L). Altogether, these results suggest that the dorsal aortae calibre and lumen reduction seen in the *Dll4*^-/- ^embryos is mainly caused by the increased outward migration of the endothelial cells from the central dorsal aortae to peripheral regions of the embryo. As *Dll4 *is not expressed in non-vascular tissues at these stages of development, the observed phenotype suggests a specific increase of endothelial cell response to normal angiogenic attractant gradients produced by surrounding tissues.

### Venous localization of the *Dll4*^-/- ^"arterial" endothelial cells

**Figure 4:**
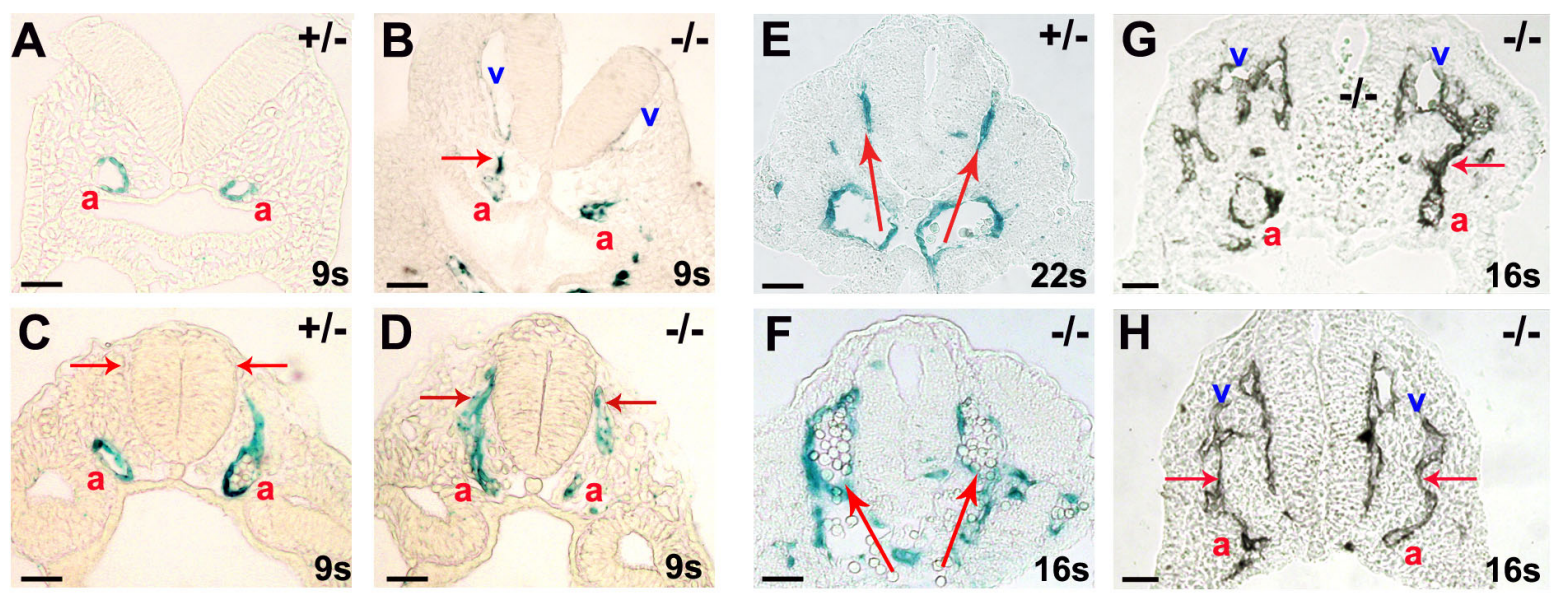
***Dll4*^-/- ^endothelial cells migrate dorsally towards the neural tube, fusing with developing anterior cardinal veins**. X-gal (A-F) and anti-PECAM-1 (G, H) staining of cryosections from the anterior (A, B) and middle (C-F) region of the *Dll4*^+/- ^and *Dll4*^-/- ^embryos with 8.5 (A-D) and 9.25 dpc (E-H). (**A-D**) *Dll4*^-/- ^arterial endothelial cells migrate (arrows in B and D) towards the dorso-lateral region of the neural tube. In the anterior region of the *Dll4 *null embryos, the aortic (a, β-galactosidase+) cells migrate towards the developing anterior cardinal vein (v), before the onset of circulation (B). (**E, F**) At later stages, in the middle region of the embryo, this dorsal migration leads to the delocalization of the dorsal aortae. The main circulation in the mutant embryos occurs through two vessels that localize laterally (F) and not ventrally (E) to the neural tube. (**G, H**) In the anterior region of the later-stage *Dll4*^-/- ^embryos the fusion (arrows) between the dorsal sprouting of the aortae (a) and the anterior cardinal veins (v) is evident. (Scale Bars: 50 μm).

One of the distinguishable features of mammalian arterial and venous early development is that the venous angioblasts migrate and form the initial anterior cardinal vein in a dorsal position close to the neural tube, whereas arterial angioblasts form the dorsal aortae ventrally and close to the endoderm. Little is known about the molecular mechanisms that regulate this early segregation of arterial and venous lineages. In the *Dll4*^-/- ^embryos we can follow the migration of arterial endothelial cells by analysis of *lacZ *expression, because the β-galactosidase protein is very stable. In the anterior, asomitic region, of the *Dll4*^-/- ^embryos abnormal migration of "arterial" endothelial cells to the region where the primordial anterior cardinal veins form was very frequently observed (Fig. 4A–D). This migration is frequently associated with arterial-venous fusions (Fig. 4G, H) and an increase of the pool of venous endothelial cells. At later stages, in the middle (somitic) region of the embryo, almost all arterial endothelial cells localize dorso-laterally to the neural tube and not ventrally as in the wild-type embryo (Fig. 4E, F). The observed migration and delocalization of the arterial *Dll4*^-/- ^endothelial cells to the dorsal and venous region can be related with the incomplete differentiation of the "arterial" endothelial cells and abnormal increased responsiveness to the angiogenic factors produced by the neural tube. Alternatively, these cells can be less sensitive to the existing repulsive factors. We previously showed that these *Dll4*^-/- ^"arterial" endothelial cells express the vein marker *Ephb4 *and fail to express detectable levels of the arterial marker *Efnb2*. So these uncommitted "arterial" endothelial cells may respond to the distribution of the gradients like the venous angioblasts or endothelial cells.

### Arterial *Dll4*^-/- ^endothelial cells express more VEGFR-2 and Robo4 and less Endoglin

**Figure 5:**
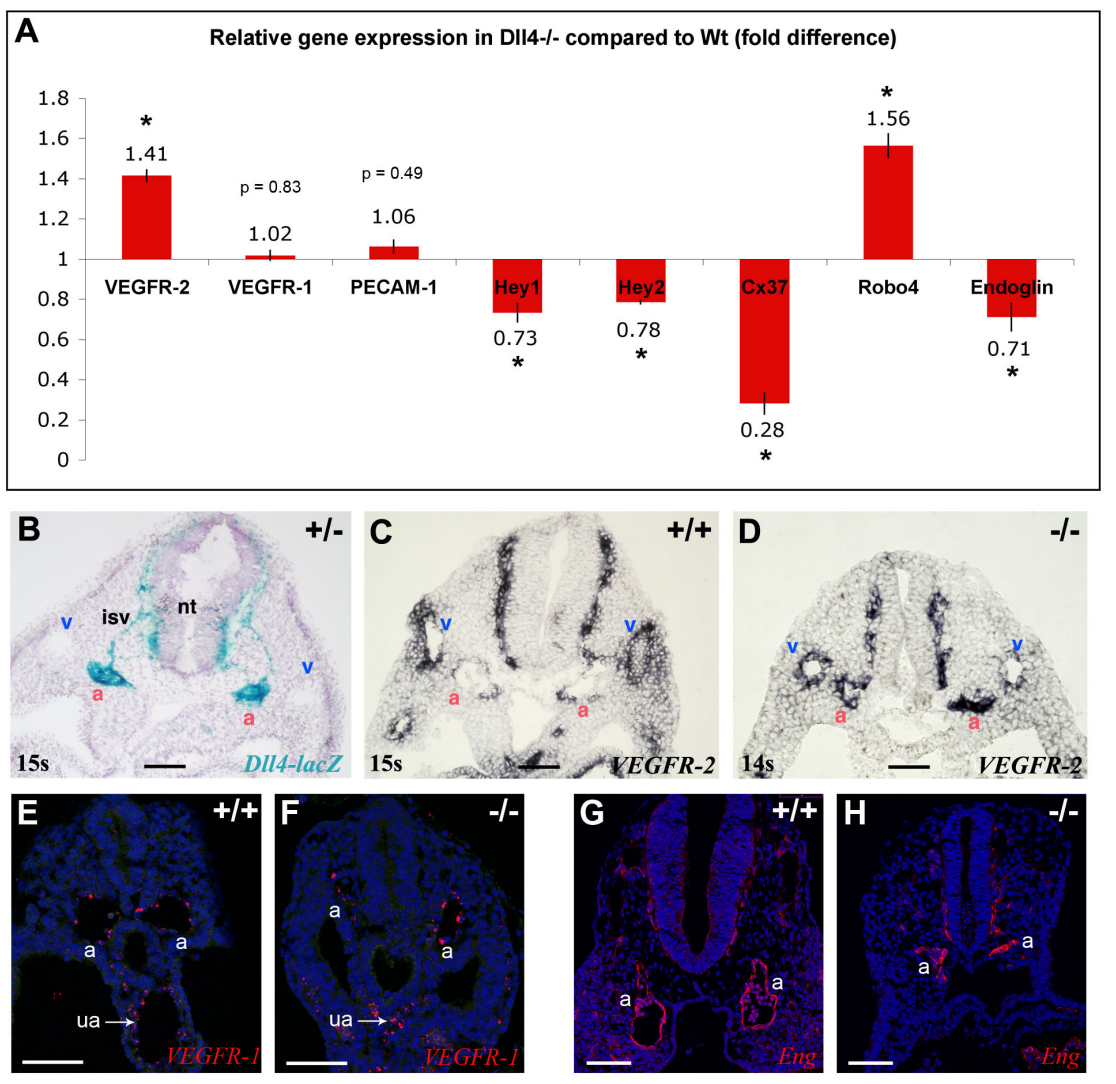
**Higher expression of *VEGFR-2 *and *Robo-4 *but lower expression of Endoglin in *Dll4*^-/- ^embryos**. (A) Relative quantitative gene expression in *Dll4*^-/- ^embryos compared to *Dll4*^+/+ ^(n = 4 per group) show increased expression of VEGFR-2 and Robo-4 and lower expression of Endoglin in the *Dll4*^-/- ^mutants. Values were normalized to those of β-actin. (**B-D**) *In situ *hybridization on 9.0 dpc embryos cryosections show upregulation of VEGFR-2 specifically in *Dll4*^-/- ^dorsal aortae (a). (B) In a thick section (50 μm) of a *Dll4*^+/- ^embryo strong expression of Dll4 can be observed on the dorsal aortae and intersomitic vessels but no expression is detected on veins (v). (C) In a *Dll4*^+/+ ^embryo VEGFR-2 is highly expressed in the veins and weakly expressed on the Dll4 expressing dorsal aortae. (**D**) In the absence of the arterial *Dll4*, VEGFR-2 is expressed at the same or at a higher level in the dorsal aortae compared to the anterior cardinal veins. (**E, F**) *VEGFR1 (Flt1) in situ *hybridization (in red) showing similar endothelial expression levels between control and mutant aortae (a) and umbilical arteries (ua). DAPI in blue. (**G, H**) Imunostaining for Endoglin (CD105) showing similar protein levels in endothelial cells of control and mutant embryos. Error bars represent st.dev. * indicates p < 0.05. (Scale Bars: 100 μm).

**Figure 6:**
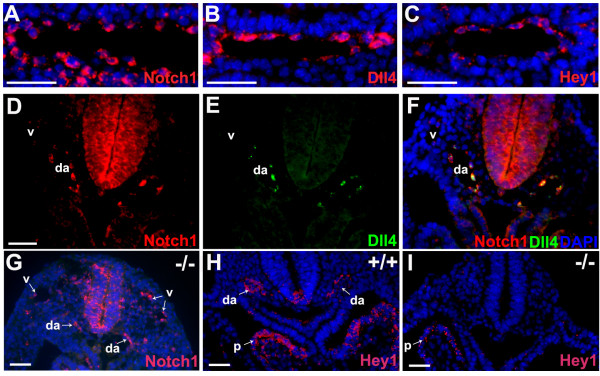
**Notch1 and *Dll4 *are expressed at different levels in different cells but at high levels in the same cell of the developing arteries**. (**A-C**) *In situ *hybridization on consecutive cryosections of 8.75 dpc (12 ss) *Dll4*^+/+ ^embryos showing that *Notch1*, *Dll4 *and *Hey1 *are expressed (red) in almost all aortic endothelial cells. (**D-F**) Double *in situ *hybridization for *Notch1 *and *Dll4 *reveals that these genes are expressed at high levels in the same cells. Note also that *Notch1 *is weakly expressed on the anterior cardinal veins (v), where there is no expression of *Dll4*. (**G-I**) In the *Dll4*^-/- ^embryos the Notch1 receptor is still expressed on dorsal aortae (da) but there is no detectable endothelial expression of the effector Hey1 (I), although its expression remains in the pericardium (p). (Scale Bars: 50 μm).

To better understand the molecular mechanisms that can be at the origin of this abnormal behaviour of the *Dll4*^-/- ^endothelial cells we performed quantitative RT-PCR for several known early angiogenesis regulator genes. The analysis was done with total RNA extracted from 11 ss embryos. This stage was selected because the effects of the mutation and defective blood flow in remodelling are just starting and the expression of known arterial markers is increased comparative to earlier stages. The use of total RNA from the embryos limited our ability to determine the expression changes specifically within endothelial cells. The results show a 41% increase in the *VEGFR-2 *expression level in the *Dll4*^-/- ^embryos and no significant variation of *VEGFR*-1 expression. In addition, *Robo4 *was upregulated by 56% and *Endoglin *downregulated by 41% (Fig. 5A). As the *Dll4 *gene is only expressed in the embryonic arteries, whereas *VEGFR-2*, *Robo4 *and *Endoglin *are also expressed in angioblasts and venous endothelial cells [17,23-25], this difference could be even more significant if RNA from only arterial endothelial cells had been used. The *PECAM*-1 expression level was used as a control of the endothelial fraction in the samples and *Cx37*, a known arterial specific marker, was used as a control of arterial differentiation. Its expression is reduced almost four-fold in the *Dll4*^-/- ^embryos. As expected, the canonical Notch effectors *Hey*1 and *Hey*2 are also downregulated in the mutants. The reduction in the expression level is probably not higher because these two genes are also expressed in other non-endothelial tissues. By *in situ *hybridization on *Dll4*^-/- ^sections we did not detect *Hey1 *mRNA in the arterial endothelial cells (Fig. 6I). On the other hand, the levels of expression of *Tie*-2 and *VE-cadherin *genes showed no significant differences between wild-type and *Dll4*^-/- ^embryos (data not shown).

Since these qRT-PCR results represent differences in whole embryo expression levels, *in situ *hybridization with a probe for the *VEGFR*-2 mRNA on wild-type and *Dll4*^-/- ^embryos was carried out. In wild-type and heterozygous embryos the *VEGFR*-2 gene was found to be more strongly expressed in the venous endothelium than in the arterial endothelial cells, where the *Dll4 *gene is strongly expressed (Fig. 5B, C). Interestingly, in the *Dll4*^-/- ^embryos the expression level of *VEGFR*-2 in arterial endothelial cells is higher and similar to its expression in veins (Fig. 5D). *In situ *hybridization for *VEGFR*-1 and imunostaining for endoglin was carried out without detection of any clear difference between *Dll4*^+/+ ^and *Dll4*^-/- ^aortae. Together these results show that, in relation to their wild-type counterparts, *Dll4*^-/- ^endothelial cells express more *VEGFR*-2 and *Robo4*, both of which are involved in the regulation of endothelial cell proliferation and migration [23,26-28] and express less the TGF-β accessory receptor endoglin, which when ablated *in vitro *increases endothelial cell proliferation [29] and *in vivo *decreases arterial stability and smooth muscle cell recruitment [30].

### Notch-*Dll4 *signalling in developing arteries does not occur through lateral inhibition

Notch signalling, in most cellular processes studied so far, functions through lateral inhibition, where a Notch signal receiving cell upregulates the expression of the Hes/Hey genes and downregulates the expression of Delta. This cell can no longer activate the pathway in neighbouring cells (that will in turn express more Delta) and therefore will acquire a different fate. This mechanism allows the differentiation between initially equivalent cells.

To understand how the Notch pathway regulates endothelial cell differentiation, we analysed endothelial cell expression of *Notch1*, *Dll4 *and *Hey1 *in early development of the arteries (dorsal aortae). In the initial stages of arterial development, *Dll4 *expression is patchy and not detected in all endothelial cells (data not shown). Some hours later, its expression is present in almost all endothelial cells of the dorsal aortae, although the level of expression differs from cell to cell. The expression of *Notch1 *and the effector *Hey1 *is also found in almost all arterial endothelial cells (Fig. 6A, B and 6C), which suggest that these genes can be expressed in the same cells. To explore this possibility, double *in situ *hybridization for *Notch1 *and *Dll4 *mRNA was carried out, showing that these genes are indeed expressed at high levels in the same cell (Fig. 6D, E and 6F). These results indicate that Notch activation in the arterial endothelium by Dll4 is likely to be inductive rather than inhibitory. *Notch1 *expression is also detected in the cardinal vein endothelium at early stages (Fig. 6D), but here it is lower than in the dorsal aortae, probably due to lack of Notch signalling induction by *Dll4*. In fact, *Hey1 *expression was not detected in the venous endothelium even at earlier stages of vein development (data not shown). In *Dll4*^-/- ^embryos, *Notch1 *expression was still detected in both veins and dorsal aortae, but in these embryos the expression levels seem to be equal between dorsal aortae and cardinal veins (Fig. 6G), suggesting that there is always a basal level of *Notch1 *expression that is independent of Dll4, and that Dll4 further induces *Notch1 *expression levels. Furthermore, there is a basal expression of *Dll4 *that is independent of Notch1 activation, since the *Dll4*-lacZ reporter gene is still expressed in *Dll4*^-/- ^embryos in the absence of *Hey1 *expression (Fig. 6H, I).

## Discussion

**Figure 7:**
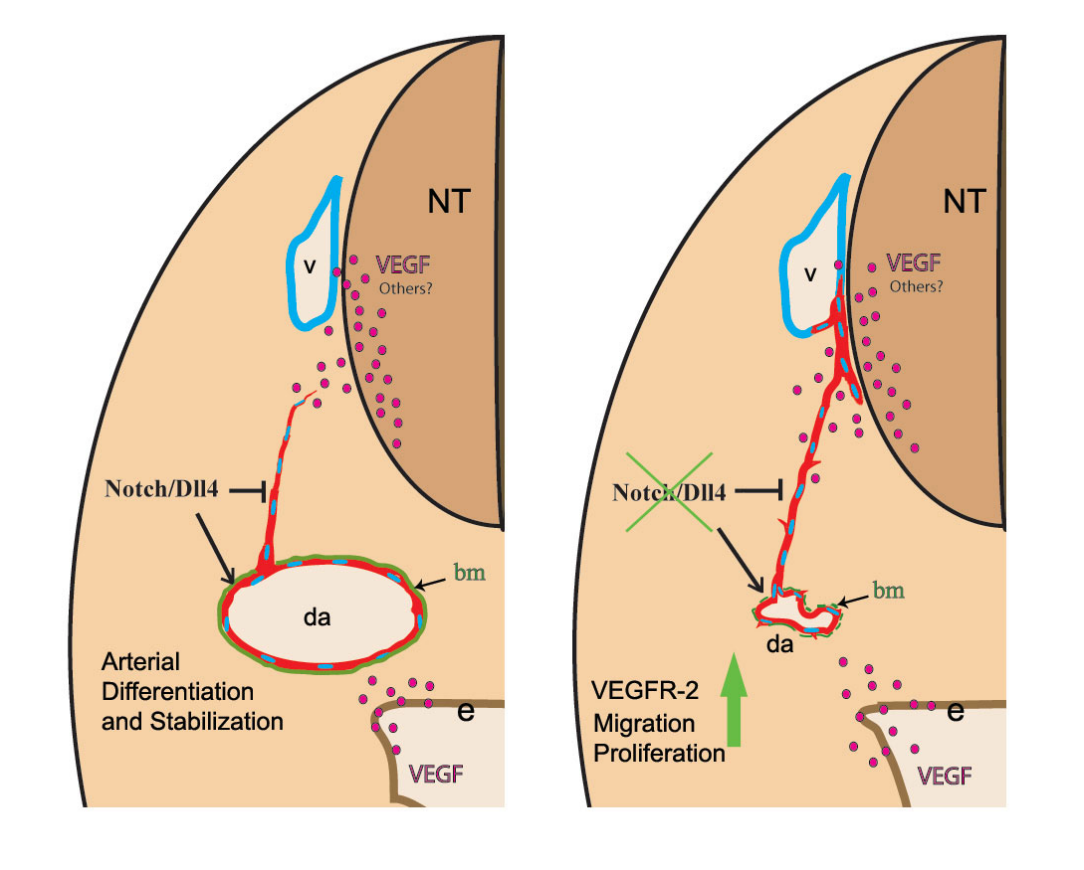
**Regulation of arterial calibre and endothelial cell migration by Dll4**. Model illustrating the difference in the behaviour of endothelial cells in the presence or absence of Notch signalling mediated by the *Dll4 *ligand in the early embryo arterial endothelium. The loss of Notch signalling leads to downregulation of arterial specific genes and upregulation of VEGFR-2 which may increase migration and proliferation of endothelial cells. The dorsal aortae (da) endothelial cells migrate towards the lateral region of the neural tube (nt) or pre-formed veins (v) with which they fuse. In the *Dll4*^+/+ ^embryos Notch signalling induces arterial endothelial cell differentiation and stabilization, as well as promoting the formation of a consistent basement membrane (bm).

The initial steps of arterial morphogenesis require the migration of endothelial precursors and their assembly into lumenized vessels. Shortly after these vessels grow and branch accordingly to the cues provided by the surrounding tissues. Previous studies with Notch signalling defective zebrafish and mouse embryos showed the importance of the pathway in early arteriogenesis [11,31]. Here we show that the Notch ligand Dll4 regulates the arterial calibre and lumen by modulating the endothelial cell response to angiogenic factors like VEGF (Fig. 7).

The *Dll4 *gene is strongly expressed in the primordial arteries and heart of early embryos and its complete loss of function causes early and severe vascular defects [15]. As disruption of heart morphogenesis in several mutants is coincident with defects in vascular development, it is sometimes difficult to discern if failure of perfusion led to defective vascular development, or whether the vascular developmental defect is an independent event. Furthermore, in the early embryo it has previously been shown that blood flow can regulate the expression of some arterial specific genes, like *Efnb2 *[21], which is not detected in *Dll4*^-/- ^arterial endothelial cells [15]. To clarify if the early phenotype is caused by endothelial or cardiac/blood flow defects, *Dll4*^-/- ^embryos were analysed before the onset of circulation. The results indicate that the arterial calibre reduction in these mutants is already clear at 8 ss, thus occurs earlier than the establishment of perfusion, and therefore cannot be caused by impaired haemodynamic forces but constitutes a primary vascular defect. This very early remodelling defect suggested a role of *Dll4 *in early vasculogenesis. However, the expression analysis of *Notch1*, *Dll4 *and *Hey1 *in early embryos revealed that only *Notch1 *can be expressed in angioblasts, which excludes the involvement of the Dll4 ligand in the initial endothelial cell differentiation from mesodermally derived cells.

*Jagged1*(*Jag1*) is another Notch ligand that is also expressed in the early embryo arterial endothelium. Total or endothelial specific *Jag*1 loss of function leads to embryo death at 10.5 dpc [32,33], one day later than loss of Notch1 or Dll4. Endothelial *Jag1 *mutants show defects in the development of the arterial vascular smooth muscle without having a decrease of Notch signalling in endothelial cells [32]. Our results show that the *Dll4*^-/- ^embryos suffer severe arterial calibre reduction before the existence of clearly differentiated smooth muscle cells (α-SMA+). Furthermore the mutant aortas are surrounded by neural crest derived smooth muscle cell progenitors (SM22α+), some of which are associated with the reduced mutant arterial endothelium. However these results do not exclude a role of Dll4 in arterial smooth muscle development at later stages of development.

The morphologic analysis of early (6–10 ss) dorsal aortae shows that the arterial calibre of the *Dll4*^-/- ^embryos is much smaller than that of the wild-type, even though the number of endothelial cells is only slightly reduced. In addition, there is lack of a uniform laminin-rich basement membrane under the endothelial cells and a defective deposition of endothelial matrix proteins like collagenIV and specially fibronectin. Thus the abnormal organization of the mutant endothelial cells can be partially explained by defects in the formation of a proper endothelial basement membrane. These defects could be the result of either reduced production or active degradation of the basement membrane components. One of the most obvious consequences of this arterial cohesion defect is the extensive haemorrhaging observed in these mutant embryos upon the onset of blood flow. Interestingly, the defect observed in the basement membrane occurs in both the reduced and normal calibre regions of the *Dll4*^-/- ^dorsal aortae. This suggests that the arterial calibre reduction is not solely caused by a weaker and incomplete, and thus permissive, basement membrane, although this may influence the behaviour of endothelial cells in the presence of adequate angiogenic stimuli. In fact, the *Dll4*^-/- ^dorsal aortae present a normal calibre only in the presomitic region, where VEGF is produced exclusively by the endoderm. In the anterior and middle somitic regions of the embryo, VEGF is also produced by the neural tube and somites, forming a concentration gradient within the newly formed intersomitic spaces, and this difference in VEGF production may underlie the difference in arterial diameter between the anterior and posterior regions of the embryo. Besides VEGF, other neural tube derived factors like slits, semaphorins and netrins can attract endothelial cells and be responsible for the correct alignment of the dorsal aortae, since ablation of VEGF in the peripheral nerves of the skin does not impair the co-alignment between arteries and nerves [34].

The *Dll4*^-/- ^aortic endothelial cells are likely to respond more than their wild-type counterparts to the neural tube or somite secreted factors, because they migrate earlier and in higher numbers. Alternatively these mutant endothelial cells could be less susceptible to the repulsive factors produced in neural tube or somites. This is unlikely, as vascular invasion of somites or neural tube was not observed. Besides increased migration, the mutant arterial endothelial cells have increased proliferation to nearly twice that of wild-type endothelial cells, which further confirms the higher responsiveness of these cells to local angiogenic factors. These results indicate that the observed smaller arterial calibre and reduced number of endothelial cells is caused by an active outward cell migration that overcomes the increase in arterial cell proliferation. Previous *in vitro *studies with human endothelial cells also demonstrated that Notch signalling activation decreases endothelial cell proliferation [35,36]. Our *in vivo *findings are consistent with these observations and suggest that Dll4 induced Notch signalling can be a physiological inhibitor of endothelial cell proliferation, allowing cells to differentiate and form an optimal vascular lumen.

The role of Notch signalling in early angiogenesis appears therefore to be fundamentally different than that described in Notch regulation of early neurogenesis, in which a pool of non-committed neuroblasts is induced to proliferate and prevented from differentiating by Notch controlled lateral inhibition [37,38]. The *in situ *hybridization data in the current study suggests that arterial endothelial Notch signalling is inductive rather than inhibitory because the Notch1 receptor and the Dll4 ligand are expressed at high levels in the same cell and almost in all aortic endothelial cells. The *in vivo *activation of Notch signalling in adult mouse arteries leads to increased expression of the ligand Dll4, Notch receptors, and downstream effectors such as bHLH transcription factor Hey1, which supports the possibility that Notch signalling induces a positive loop in endothelial cells resulting in induction of Notch receptor and its cognate ligand [39]. Furthermore there is a basal level of Notch1 and Dll4 expression in arterial endothelial cells that is independent of Notch-Dll4 signalling, since there is expression of both the *Dll4*-*lacZ *reporter and *Notch1 *in the *Dll4*^-/- ^mutants.

The RT-PCR and *in situ *hybridization results obtained are consistent with the observed mutant endothelial cell behaviour. The *Dll4*^-/- ^arterial endothelial cells express more VEGFR-2 which is required for VEGF mediated endothelial cell migration and proliferation [40]. This direct relationship between VEGF signalling and the Notch pathway was previously suggested by *in vitro *studies with HUVECs where Notch activation, through the upregulation of Dll4, reduces the expression of VEGFR-2 and Nrp-1 [41,42]. The receptor Robo-4 is also upregulated in the *Dll4*^-/- ^embryos. At the stages analysed it is expressed throughout the endothelial network and, like VEGFR-2, it is further induced at sites of active angiogenesis. *In vivo *knockdown of *Robo-4 *in zebrafish embryos suppresses the formation of intersomitic vessels due to inhibition of endothelial cell sprouting and migration, seen by time-lapse analysis [23], which is the opposite to the phenotype observed in *Dll4*^-/- ^mouse embryos. Robo-4 also participates in attraction-guidance through Rho-GTPases in vascular development, as *Robo-4 *gain of function experiments *in vitro *showed activation of Cdc42 and Rac1 Rho-GTPases [26]. Therefore Robo-4 may be one of the mediators of the increased migration of *Dll4*^-/- ^endothelial cells in response to its ligand Slit produced by the somites and the neural tube. Interestingly, *Robo-4 *levels decline in the *Dll4 *expressing dorsal aortae after 9.0 dpc [24] and in the mouse retina *Robo-4 *is not expressed in the tip cells known to highly express *Dll4 *[28]. However, deletion of *Robo-4 *in the mouse is not lethal and developmental angiogenesis occurs normally [28], suggesting that its function might be compensated by other genes. Nonetheless its loss of function enhances post-natal induced pathologic angiogenesis and endothelial permeability. This inhibition is thought to depend on Slit2-Robo4 suppression of VEGF165-Src induced endothelial migration and tube formation but not proliferation [28]. Although controversial these results suggest that Robo4 could act differently during development and pathological angiogenesis. We also found a significant reduction in the expression of the TGF-β1 co-receptor endoglin. *Eng*^-/- ^mutant embryos die around 10.5 dpc due to several cardiovascular defects that also include abnormal arterio-venous fusions, and this phenotype is independent of loss of *Efnb2 *expression [30,43]. Furthermore, *in vitro *studies show that *Eng*^-/- ^endothelial cells have increased proliferation [29].

The vessel response to angiogenic factors must be tightly regulated to ensure a balance between the formation of new sprouts and the remodelling of previously formed vessels. Such regulation allows the formation of a hierarchical system of vessels with critical contribution from the Notch-Dll4 pathway, which modulates VEGF signalling in endothelial cells. An important example of this role has been recently reported in relation to the vascularisation of the retina in newborn mice [44-46].

We observed migration of *Dll4*^-/- ^aortic endothelial cells towards the developing anterior cardinal veins in the dorso-lateral region of the neural tube (Fig. 7). This migration and delocalization of the "arterial" *Dll4*^-/- ^endothelial cells to the assembly region of the primordial veins may be related to faulty differentiation [13,15,31,47] and increased responsiveness to the angiogenic factors.

VEGF induces expression of Dll4 [48] which in turn, by activating the Notch pathway, reduces endothelial cell sensitivity and responsiveness to VEGF. We thus suggest that the Notch pathway could constitute a negative feedback mechanism to ensure that the formation and extension of new arterial vessel branches occurs only after the growth and stabilization of the preceding main arterial vessels. Without Notch signalling the arterial endothelial cells proliferate and migrate in an uncontrolled manner, resulting in the formation of a vasculature that is not efficient in blood distribution due to exaggerated branching and extension of the preformed vessels. A number of recent reports indicate that this is also the case during tumour neoangiogenesis, with loss of Notch activation by Dll4 resulting in an inefficient vascular bed due to a disproportionate increase in vessel branching [49-51].

## Conclusion

Many of the currently used anti- or pro-angiogenic therapies were designed after important findings concerning early mouse embryo vascular development. The present data shows that activation of the Notch pathway by the ligand Dll4 can regulate several different key steps in arteriogenesis through interaction with other important endothelial signalling mechanisms. The understanding of these interactions between different signalling pathways offers alternatives for future therapeutic intervention.

## Authors' contributions

RB participated in the design of the studies, acquisition of the data, preparation of the figures and writing of the manuscript. AT participated in the experiments discussion, improved some techniques and revised the manuscript. MH carried out the first experiments which originated this research work and revised the manuscript. DH, LLC, JR and PSG participated in the discussion of the experiments and revision of the manuscript. AD conceived the study, participated in its design and coordination and helped write the manuscript. All authors read and approved the final manuscript.
